# Critical View of Safety in Laparoscopic Cholecystectomy: A Word of Caution in Cases of Aberrant Anatomy

**DOI:** 10.1055/s-0042-1744154

**Published:** 2022-10-18

**Authors:** Maria Ioanna Antonopoulou, Dimitrios K. Manatakis

**Affiliations:** 1 Department of Surgery, Athens Naval and Veterans Hospital, Athens, Greece

**Keywords:** anatomy, gallbladder, laparoscopic cholecystectomy, vasculobiliary injury

## Abstract

**Introduction** To avoid vasculobiliary injuries, the Critical View of Safety (CVS) technique is strongly recommended during dissection of the hepatocystic triangle. It entails three basic steps as follows: (1) complete clearance of the hepatocystic triangle of fibrofatty tissue, (2) separation of the lower part of the gallbladder from the cystic plate, so that (3) two and only two structures are seen entering the gallbladder.

**Case History** In this video vignette, we present the case of an aberrant hepatic artery, coursing subserosally parallel to the gallbladder wall. Despite presumably achieving all three CVS requirements, the surgeon did not proceed to clipping and dividing the two structures, preventing a major vascular injury. Due to its unusually large caliber, the artery was carefully dissected, and multiple smaller branches to the gallbladder were ligated instead, until it was definitively identified entering into the hepatic parenchyma of segments IVb–V.

**Discussion** The CVS approach was originally conceived as a means for the conclusive recognition of the cystic duct and artery to prevent misidentification errors. However, in such cases of extreme anatomical variations, the CVS may indeed have certain limitations. Therefore the surgeon should always maintain a high degree of suspicion and a low threshold for alternative bail-out options.

Major vasculobiliary injuries during laparoscopic cholecystectomy continue to occur at 0.2 to 0.6% and the vast majority (up to 85%) are related to misidentification of anatomical structures. 1 The Critical View of Safety (CVS) was described in 1995 as a target identification method and has the following three requirements: (1) clearance of the hepatocystic triangle of all fibrofatty tissue, (2) two and only two structures are seen connected to the gallbladder, and (3) the lower third of the gallbladder is dissected off the cystic plate. 2 When all three criteria are met, the two tubular structures are securely identified as the cystic duct and cystic artery and can be safely divided.

However, the CVS approach may have certain limitations in cases of extreme anatomical variations. In this video vignette, we present the case of an aberrant right hepatic artery coursing parallel to the gallbladder wall which could result in a vascular injury, despite achieving a critical view ( Video 1 ; available in the online version).


**Video 1**


## Case Presentation

A 28-year-old, otherwise healthy, Caucasian male was scheduled for elective laparoscopic cholecystectomy due to symptomatic cholelithiasis (biliary colic). The operation was performed by a consultant surgeon with experience of >200 cases of laparoscopic cholecystectomy who routinely applies the CVS approach. As per standard department policy, the completed CVS was documented by video recording, prior to division of any critical structures.

**Fig. 1:**
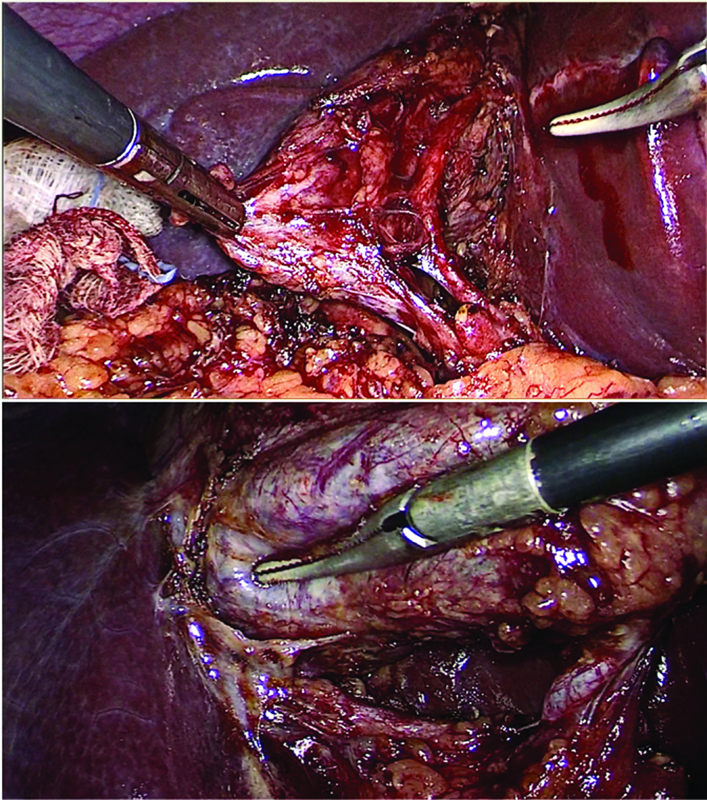
Anterior and posterior view of the presumed Critical View of Safety.

**Fig. 2:**
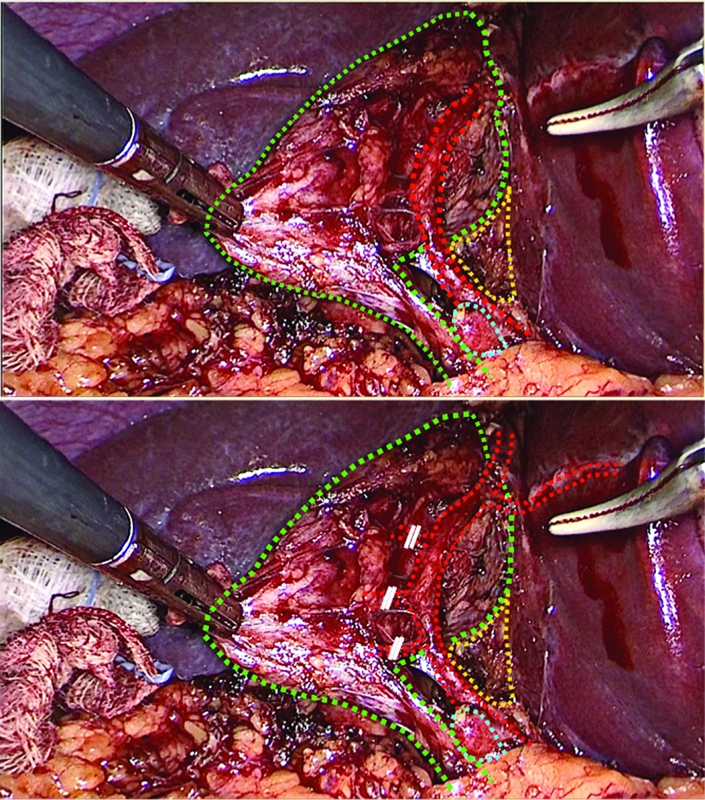
Initially purported the Critical View of Safety (above) versus definitive anatomy (below) (green: gallbladder and cystic duct, red: cystic artery and aberrant right hepatic artery, blue: cystic lymph node, yellow: lower part of cystic plate).

On initial inspection of the hepatocystic triangle, a vessel putatively recognized as the “cystic artery” was observed subserosally, parallel to the gallbladder wall. After complete clearance of the triangle and partial mobilization of the lower part of the gallbladder off the liver bed, only two structures were identified entering the gallbladder ( Fig. 1 ). However, during the team time-out, the purported “cystic artery” was noticed to be of unusually large caliber and with a course possibly reentering into the hepatic parenchyma of segments IVb–V. Therefore, instead of clipping the main vessel, the surgeon proceeded to ligation of multiple fine branches to the gallbladder wall, preserving the main arterial trunk ( Fig. 2 ). This was carefully dissected and eventually identified as an aberrant hepatic artery, possibly the right anterior hepatic artery. Subsequently, the cystic duct was also clipped and divided and the gallbladder was taken off the liver bed. The patient was discharged on the following day and had an uneventful postoperative recovery.

## Discussion

The vascular anatomy of the hepatocystic triangle poses a challenge to every surgeon performing laparoscopic cholecystectomy. Typically, the cystic artery branches off the right hepatic artery and courses in the triangle. 3 4 However, both the origin and the course of the cystic artery may be highly variable, and in up to 20% of cases, the cystic artery is not found within the anatomical boundaries of the triangle. 3 Interestingly, a replaced right hepatic artery has been described coursing close to the gallbladder wall and giving off numerous small branches, such that on laparoscopy, it may appear as a particularly large cystic artery, predisposing to injury. 3

By definition, the idea behind the CVS was conceived precisely to prevent such anatomical traps due to misidentification. 2 5 Its rationale is based on a two-step approach. After clearance of the hepatocystic triangle, the two tubular structures are putatively identified as the cystic duct and artery. When the lower part of the gallbladder is mobilized off the cystic plate, then these two structures are undoubtedly demonstrated to be the cystic structures. 6

Yet, despite its solid theoretical basis, achieving the CVS does not altogether prevent major vasculobiliary injuries. Large case series of laparoscopic cholecystectomies, in which the CVS was routinely applied, have reported rates of major bile duct injuries up to 0.54%. 7 8 9 Regarding vascular injuries on the other hand, evidence is scarce. An old study revealed a rate of 0.25% for vascular injuries, with the right hepatic artery being by far the most commonly injured vessel (>90%). 10 11 Accidental ligation of a hepatic artery may cause clinically significant liver ischemia in up to 10% of patients, leading either to rapid necrosis, abscess formation or liver atrophy. 11 In cases of combined vasculobiliary injury, bile duct ischemia could result in early anastomotic leakage or manifest late, as stenosis of the biliodigestive anastomosis. 11

In our patient, the two “red flags” were the relatively large caliber, to what would normally be expected for the cystic artery, and its course, which gave the impression of not ending on the gallbladder wall but rather continuing anteriorly toward segments IVb–V. Two similar cases have been described in the literature. In the case report by Yamazaki et al, preoperative computed tomography (CT) scan identified the right anterior inferior branch for segment V arising from the left hepatic artery and travelling across the neck of the gallbladder. The procedure was concluded without complications by the fundus-first approach. 12 In the case report by Blecha et al, an aberrant right hepatic artery was identified intraoperatively, adherent to the anterior surface of the gallbladder. The cystic artery branched off laterally over the gallbladder fundus. After gallbladder removal, the aberrant artery was visible on the gallbladder bed, entering the liver at an unusually anterior location. 13

Therefore the surgeon should always maintain a high degree of suspicion and a low threshold for bail-out alternatives. 14 The two alternative options discussed by the surgical team over the time-out were laparoscopic fundus-first cholecystectomy and conversion to the open approach, in case the dissection could not proceed safely. Intraoperative imaging alternatives may include laparoscopic ultrasound or indocyanine green fluoroscopic angiography. 15 16 However, these modalities are not readily available in most hospitals and require expertise in the interpretation of the images. Precise characterization of the origin and course of the aberrant vessel in our patient would only be feasible by angiography or CT scan; however, this was not indicated preoperatively.

In conclusion, we firmly believe that the CVS is a powerful tool and should belong to the armamentarium of every modern surgeon. However, it has its limitations in cases of certain anatomical variations. It should be part of an overall “culture of safety” in laparoscopic cholecystectomy that should combine profound knowledge and understanding of the relevant anatomy and mechanisms of vasculobiliary injuries, proper surgical technique, situational awareness of potential error traps, and liberal use of bail-out options in difficult cases. 17 18 19 20
